# Early experience with Mini-CEX in otorhinolaryngology residency: feasibility, feedback quality, and perceived educational impact

**DOI:** 10.1016/j.bjorl.2026.101944

**Published:** 2026-10-31

**Authors:** Andrea Arantes Braga, Natalia Shigematsu, Carolina Sponchiado Miura, Denny Marcos Garcia, Valdes Roberto Bollela, Fabiana Cardoso Pereira Valera, Camila de Giacomo Carneiro

**Affiliations:** a Universidade de São Paulo, Faculdade de Medicina de Ribeirão Preto, Department of Ophthalmology, Otorhinolaryngology and Head and Neck Surgery, Division of Otorhinolaryngology, Ribeirão Preto, SP, Brazil; b Universidade de São Paulo, Faculdade de Medicina de Ribeirão Preto, Internal Medicine Department, Tropical and Infectious Diseases Division, Ribeirão Preto, SP, Brazil

**Keywords:** Clinical competence, Education, Medical, Otorhinolaryngology, Feedback

## Abstract

**Objectives:**

To describe the implementation of the Mini-Clinical Evaluation Exercise (Mini-CEX) in an Otorhinolaryngology residency program and to analyze assessment outcomes, feedback, and residents’ and preceptors’ perceptions during the first 21 months.

**Methods:**

This retrospective observational study with a cross-sectional quantitative survey and brief open-ended responses comprised a retrospective review of Mini-CEX forms completed between January 2022 and October 2023 in the Otorhinolaryngology outpatient clinics of Hospital das Clínicas da Faculdade de Medicina de Ribeirão Preto da USP (HCFMRP/USP). Assessments were summarized by subspecialty, complexity, training year, and preceptor experience (≤ 10 vs. >10 years). Participants answered a 27-item online questionnaire on Mini-CEX knowledge and perceptions, plus three open-ended questions.

**Results:**

Forty Mini-CEX encounters were completed for 22 residents by 17 preceptors. Most assessments occurred in otology (52.5%), whereas laryngology was the least represented subspecialty (2.8%). Cases were predominantly of moderate or high complexity (>60%). 46% of assessments were rated as “above expectations”, with no significant differences among assessed domains (p = 0.19). Less experienced preceptors were selected more often and assigned lower scores than more experienced preceptors (p < 0.0001); non-faculty preceptors assigned lower scores than faculty members (p = 0.0032). First-year residents scored lower than second- and third-year residents (p = 0.0014). Although 90% received feedback, only 2.5% of feedback entries met completeness criteria, regardless of preceptor experience (p = 0.72). The survey response rate was 90%. Correct responses averaged 87% for knowledge items, while agreement for impact items was 58%; preceptors reported a higher impact than residents (p = 0.006).

**Conclusion:**

Mini-CEX implementation in an Otorhinolaryngology residency program was feasible and captured the expected progression by training year; however, it revealed insufficient feedback documentation and potential rating leniency. To enhance its educational impact, rater training, protected time for assessment, standardized feedback, and broader subspecialty sampling are recommended.

**Level of evidence:**

IV.

## Highlights

• Mini-CEX was feasible in a busy ENT outpatient residency setting.
• Scores improved across residency years, reflecting expected training progression.
• Improving feedback quality and protected time may enhance Mini-CEX impact.

## Introduction

The Mini-Clinical Evaluation Exercise (Mini-CEX) is one of the most widely used Workplace-Based Assessment (WBA) tools in medical education.^1^ By combining direct observation of authentic clinical encounters with structured feedback, it supports both assessment and learning within routine clinical practice.^2^ Its educational impact depends heavily on implementation factors such as assessor training, protected assessment time, and feedback quality.^3^, ^4^, ^5^, ^6^, ^7^

Recent literature demonstrates a sustained increase in Mini-CEX-related publications and international implementation across multiple specialties.,^8^,^9^ Despite its widespread use, the Mini-CEX remains underexplored in Otorhinolaryngology and little is known about feedback quality, perceptions, and barriers to implementation in this specialty.^10^, ^11^, ^12^, ^13^ This represents an important knowledge gap, particularly in surgical specialties such as Otorhinolaryngology.

The growing adoption of Competency-Based Medical Education (CBME) has increased the importance of workplace-based assessments, emphasizing longitudinal evaluation of residents' performance in real clinical settings.^14^^,^^15^ In Brazil, CNRM Resolution nº 4/2023 reinforced this approach by requiring continuous assessment of residents across cognitive, psychomotor, and professional domains using validated assessment methods and documented feedback.^16^

Recent studies have highlighted that the educational effectiveness of the Mini-CEX depends less on numerical ratings and more on the quality of feedback conversations generated during the assessment process.^16^, ^17^, ^18^

Therefore, this study aimed to: assess the feasibility of Mini-CEX implementation in an otorhinolaryngology residency program; evaluate the quality of feedback documentation; examine residents’ and preceptors’ perceptions regarding its educational impact; and identify barriers and opportunities for improving its implementation.

## Methods

This retrospective observational study with a cross-sectional quantitative survey and brief open-ended responses comprised (i) A retrospective review of all mini-CEX forms completed between January 2022 and October 2023 in the Otorhinolaryngology outpatient clinics of HCFMRP/USP and (ii) A cross-sectional survey of residents and preceptors regarding knowledge of institutional Mini-CEX procedures and perceived educational impact.

In our Otorhinolaryngology residency program at HCFMRP/USP, preceptors received initial training on the Mini-CEX through didactic sessions and written guidance. The tool was introduced in 2021, but early uptake was low. In 2022, the Mini-CEX was incorporated into the resident handbook, although the expected frequency of use remained loosely specified. From 2023 onward, quarterly completion became mandatory, with the aim of sampling different competencies (history taking, physical examination, and patient counseling), subspecialties, and evaluators.^1^^,^^19^ At that stage of implementation, feedback was not a major focus of the training.

Data collection was conducted from January 2022, when the Mini-CEX was incorporated into our residency program handbook, through October 2023. This period preceded the implementation of Brazilian National Medical Residency Commission (CNRM) Resolution nº 4/2023, which regulates the mandatory procedures for the assessment of medical residents in residency programs nationwide. Following the implementation of this resolution, our program introduced several modifications to the Mini-CEX process; therefore, the retrospective review was limited to the pre-implementation period to ensure methodological consistency. From 2022 onward, all preceptors and residents underwent an initial training program on Mini-CEX implementation and were therefore eligible and invited to participate in the study (convenience sample).

Although the Mini-CEX was originally developed using a nine-point rating scale,^1^ we opted to simplify the instrument to a three-point scale (“unsatisfactory”, “satisfactory”, and “above expectations”) to facilitate its use by reviewers. Although this modification may have reduced the instrument's sensitivity to detect subtle changes in performance over time, it was adopted to improve the feasibility and practicality of the assessment process.

Mini-CEX forms were reviewed for subspecialty, patient characteristics, visit type (new vs follow-up), case complexity (low/moderate/high), competency assessed (history taking, physical examination, and/or patient counseling), and ratings across the domains included in our institutional form (Table 1). Forms were stratified by residency year and by preceptor characteristics (≤ 10 vs. > 10 years of preceptorship; faculty vs non-faculty).

**Table 1:** Mini-CEX assessment form.

Mini-Clinical Evaluation Exercise (Mini-CEX)
**Evaluator:**
**Date:** ____ / ____ / ______
**Resident:**
**Patient diagnosis:**
**Patient:**
**Age:** _____
**Sex:** Male () Female ()
**Type of visit:** New case () Follow-up ()
**Case complexity:** Low () Moderate () High ()
**Assessment focus:**
Interviewing () Physical examination () Patient counseling ()

**1-Interviewing skills:**
Not applicable ()
Unsatisfactory () Satisfactory () Above expectations ()
**2-Physical examination skills:**
Not applicable ()
Unsatisfactory () Satisfactory () Above expectations ()
**3-Humanistic qualities / Professionalism:**
Unsatisfactory () Satisfactory () Above expectations ()
**4-Clinical reasoning and judgment:**
Not applicable ()
Unsatisfactory () Satisfactory () Above expectations ()
**5-Communication and counseling skills:**
Not applicable ()
Unsatisfactory () Satisfactory () Above expectations ()
**6-Organization and efficiency:**
Not applicable ()
Unsatisfactory () Satisfactory () Above expectations ()
**7-Overall clinical competence:**
Not applicable ()
Unsatisfactory () Satisfactory () Above expectations ()

Evaluator’s satisfaction with the Mini-CEX:
Unsatisfactory () Satisfactory () Above expectations ()
Resident’s satisfaction with the Mini-CEX:
Unsatisfactory () Satisfactory () Above expectations ()
Evaluator feedback:

Resident feedback:

Evaluator’s signature:

Resident’s signature:

To evaluate feedback quality, narrative comments were classified as complete when they documented (a) Specific strengths, (b) Specific deficiencies/opportunities for improvement, and (c) An actionable improvement plan. Feedback that was absent, generic, or non-specific was classified as inadequate for formative purposes (Appendix 1).^18^^,^^20^^,^^21^

A 27-item online questionnaire (Google Forms)^22^ was developed based on the recommendations established by the original developers of the Mini-CEX regarding its structure, intended use, and the educational outcomes expected from its implementation.^3^^,^^8^^,^^18^^,^^23^ Ten items assessed participants’ understanding of institutional Mini-CEX procedures/constructs and principles of use, using multiple-choice and Likert-type responses.^24^ Seventeen additional items explored participants’ perceptions of changes or improvements associated with Mini-CEX implementation. For analytic purposes, knowledge items were scored as correct or incorrect according to the guidance previously provided during preceptor training and in the resident handbook; perception items were summarized as agreement with the expected impact. Three open-ended questions explored prior exposure, perceived advantages and disadvantages, and suggestions for implementation improvement. The complete questionnaire and the corresponding correct answers are provided in Appendix 2. All participants provided written informed consent before completing the questionnaire.

Score comparisons were performed using nonparametric tests (Mann-Whitney; Kruskal-Wallis with Dunn’s post hoc), and categorical variables were analyzed using Fisher’s exact test when appropriate.

### Ethics

The study was approved by the Research Ethics Committee HCFMRP/USP (CAAE: 74911323.0.0000.5440). Before completing the questionnaire, all participants provided written informed consent through an ethics committee–approved consent form, which clearly described the measures adopted to ensure data protection, data confidentiality and participant privacy.

## Results

Between January 2022 and October 2023, 40 outpatient Mini-CEX encounters were completed, 60% (n = 24) in 2022 and 40% (n = 16) in 2023. Twenty-two residents (postgraduate years 1–3) and 17 preceptors (including six faculty members) were eligible. Three residents and two preceptors completed no assessments. Residents completed one to three encounters each, whereas preceptors completed one to nine (Table 2). The target of four encounters per resident per year was not achieved. Two preceptors (13.3%) completed six to nine encounters, accounting for 40% (n = 16) of all assessments. Four preceptors with ≤ 10-years of experience (26%) performed 52% (n = 21) of all encounters.

**Table 2:** Mini-CEX form data.

Data	Total number / Mean	Percentage / SD
Number of completed Mini-CEX forms from January 2022 and October 2023	40	
Forms completed 2022	24	60.00%
Forms completed in 2023	16	40.00%
Participating residents	19 of 22	86.30%
Participating preceptors	15 of 17	87.00%
Preceptors who performed 6–9 evaluations	2	13.33%
Total evaluations performed by these preceptors	16	40.00%
Preceptors who performed 2–5 evaluations	6	40.00%
Total evaluations performed by these preceptors	17	42.50%
Preceptors who performed 1 evaluation	7	46.67%
Total evaluations performed by these preceptors	7	17.50%
Preceptors with < 10 years of preceptorship	4	26.67%
Total evaluations performed by these preceptors	21	52.50%
Preceptors with ≥ 10 years of preceptorship	11	73.33%
Total evaluations performed by these preceptors	19	47.50%
Diagnostic hypotheses across subspecialties		
Otology	19	52.78%
Rhinology	6	16.67%
Sleep Medicine	5	13.89%
Otoneurology	3	8.33%
Pediatric Otorhinolaryngology	2	5.56%
Laryngology	1	2.78%
Stomatology/ Cranio-maxillofacial surgery/ Facial aesthetics/ Head & Neck surgery	0	0%
Patient Age	32	26
Female	22	56.41%
New cases	18	46.15%
Follow-up cases	21	53.85%
Clinical case complexity	38	
High	4	10.53%
Moderate	19	50.00%
Low	15	39.47%
Assessed competencies	40	
3/Mini-CEX	3	7.50%
2/Mini-CEX	6	15.00%
1/Mini-CEX	31	77.50%
Total number of assessed competencies	49	
Interviewing	17	34.69%
Physical examination	18	36.73%
Patient counseling	14	28.57%
Missing case information	9	22.00%

Otology accounted for most encounters (52.5%), whereas laryngology was least represented (2.8%). No Mini-CEX assessments were performed in stomatology, cranio-maxillofacial surgery, facial aesthetics, or Head & Neck surgery during the study period. Patients were 2–68 years old (mean 32 ± 25 years), 56% were female, 53.8% were follow-up visits, and case complexity was high in 10.5%, moderate in 50%, and low in 39.5%. All three competencies (history taking, physical examination, and patient counseling) were assessed in 7.5% of encounters, two competencies in 15%, and a single competency in 77.5%. Physical examination was the most frequently assessed competency (36.7%), followed by history taking (34.7%) and patient counseling (28.6%). Nine forms (22%) contained incomplete clinical information, most commonly missing the diagnostic hypothesis (n = 4), patient age (n = 2), or case severity (n = 2).

Regarding the overall Mini-CEX ratings, 0.7% of assessments were rated as unsatisfactory, 38.6% as satisfactory, and 46.1% as above expectations. with no significant differences among domains (p = 0.19; Table 3). Less experienced preceptors (≤ 10-years) assigned lower scores than more experienced preceptors (p < 0.0001), and non-faculty preceptors scored residents lower than faculty members (p = 0.0032). First-year residents scored lower than second- and third-year residents (p = 0.0014).

**Table 3:** Mini-CEX assessment results by assessed domains, preceptorship experience, and residency year.

Assessed domains	U		S		AE		Median	IQR	p-value
1- Interviewing skills	0	(0%)	14	(48%)	15	(52%)	3	2; 3	p = 0.19^a^
2. Physical examination skills	0	(0%)	11	(48%)	12	(52%)	3	2; 3	
3. Humanistic qualities/professionalism	0	(0%)	10	(26%)	29	(74%)	3	2; 3	
4. Clinical reasoning and judgment	0	(0%)	20	(56%)	16	(44%)	2	2; 3	
5. Communication and counseling skills	0	(0%)	17	(46%)	20	(54%)	3	2; 3	
6. Organization and efficiency	2	(5%)	17	(45%)	19	(50%)	2	2; 3	
7. Overall clinical competence	0	(0%)	19	(51%)	18	(49%)	2	2; 3	

Preceptors with < 10-years of preceptorship	0	(0%)	47	(37%)	80	(63%)	2	2; 3	p < 0.001^b^
Preceptors with ≥ 10-years of preceptorship	2	(2%)	61	(54%)	49	(44%)	3	2; 3	

Faculty members	0	(0%)	15	(42%)	21	(58%)	3	3; 3	p < 0.0032^b^
Preceptores	2	(1%)	93	(46%)	108	(53%)	2	2; 3	

First-year residents	2	(3%)	44	(60%)	27	(37%)	2	2; 3	p = 0.0014^a^
Second-year residents	0	(0%)	30	(39%)	47	(61%)	3*	2; 3	
Third-year residents	0	(0%)	34	(38%)	55	(62%)	3*	2; 3	

U, Unsatisfactory; S, Satisfactory; AE, Above expectations.

* Significantly different from the first-year resident group.

a Kruskal-Wallis test with Dunn’s post hoc test.

b Mann-Whitney.

Feedback was provided in 90% (36/40) of encounters. Of these, 29 (80.6%) consisted of direct feedback, 6 (16.7%) included both positive and negative points, and 6 (16.7%) included an improvement plan. However, only one entry (2.5%) met the predefined completeness criteria, which required the inclusion of strengths, deficiencies, and an improvement plan. Feedback adequacy was not associated with preceptor experience (p = 0.72). Residents provided feedback in 55% of encounters, most of which was non-specific (68%). Of these, 54% addressed the preceptor’s evaluation, 22% the resident’s self-assessment, and 18% the Mini-CEX tool.

Survey administration included an initial one-week response window followed by three reminders. The response rate increased from 46% (20/43) after the first invitation to 90% (39/43) after a second invitation. The questionnaire demonstrated excellent internal consistency (Cronbach’s alpha = 0.96, 95% CI 0.95–0.98). The 10 knowledge items showed acceptable internal consistency (Cronbach’s alpha = 0.86, 95% CI: 0.78–0.91), whereas the 17 perception items demonstrated excellent internal consistency (Cronbach’s alpha = 0.96, 95% CI: 0.94–0.98). The mean score across all 27 closed-ended items was 68.5% (Table 4). Preceptors scored higher than residents overall (p < 0.001) (Table 5), and faculty members scored higher than non-faculty preceptors (p = 0.017). No significant differences were observed between preceptors according to years of experience (p = 0.35) or among residents according to training year (p = 0.80).

**Table 4:** Google Forms responses to questions on the Mini-CEX.

Questions	SD	D	NaNd	A	SA	CR	CRR
Questions on the functioning of the Mini-CEX at our institution							
1 – Each Mini-CEX assesses the resident’s ability to perform interviewing, physical examination, or patient counseling with a real patient.	2	7	7	53	28	+	82%
2 – The Mini-CEX can assess professionalism, clinical reasoning, organization/efficiency, and overall clinical competence.	0	5	12	59	23	+	82%
3 – The resident is expected to request a preceptor to conduct the Mini-CEX assessment.	2	5	7	33	51	+	84%
4 – The resident is expected to organize their schedule to maintain the Mini-CEX assessment frequency as stipulated in the resident handbook.	2	5	10	43	38	+	81%
5 – The preceptor is expected to choose the timing, the competency, and the patient for assessment using the Mini-CEX.	7	28	17	30	15	_	45%
6 – The resident is expected to organize their assessments to complete Mini-CEX evaluations covering the three competencies (interviewing physical examination, and patient counseling), with different evaluators and across different subspecialties.	2	2	3	46	41	+	87%
7 – The resident is expected to exercise judgment in selecting the most appropriate time to request an assessment so as not to disrupt service workflow.	2	5	2	36	53	+	89%
8 – The preceptor is expected to decide whether or not to provide feedback for each Mini-CEX assessment.	38	38	10	10	2	_	76%
9 – Feedback is expected to address both positive and negative aspects, be specific, direct, and assertive, avoid personal judgments, and remain independent of case severity or complexity.	0	0	7	48	43	+	91%
10 – During feedback, the preceptor is expected to encourage initial resident self-assessment, provide guidance for improvement, and solicit feedback on their own evaluation.	0	2	7	38	51	+	89%
Total correct responses (questions 1–10)							87%

Opinions regarding changes and improvements resulting from the implementation of the Mini-CEX							
11 – The feedback provided by the Mini-CEX helps residents improve their knowledge, skills, and attitudes.	2	5	12	41	38	+	79%
12 – Mini-CEX assessments can identify gaps in the teaching-learning process and guide strategies to address these challenges.	2	3	4	51	28	+	69%
13 – Mini-CEX assessments can help residents plan their continuous knowledge acquisition, thereby supporting the development of qualified professionals.	2	10	17	51	17	+	68%
14 – The Mini-CEX improves the teaching-learning process.	2	5	28	41	23	+	64%
15 – The use of the mini-CEX should be encouraged.	2	7	23	41	25	+	66%
16 – The Mini-CEX should be replaced by another assessment method.	10	28	19	7	5	_	38%
17 – The Mini-CEX is a practical assessment tool.	0	10	17	59	12	+	71%
18 – The Mini-CEX is an easy-to-use assessment tool.	2	7	12	61	15	+	76%
19 – The Mini-CEX is an efficient assessment tool.	2	10	25	48	12	+	61%
20 – The use of the Mini-CEX contributes to improving the resident-preceptor relationship.	2	15	30	41	10	+	51%
21 – The use of the Mini-CEX helps residents prepare for theoretical examinations.	17	35	25	10	10	_	52%
22 – The use of the Mini-CEX influences the development of professionalism among residents.	5	12	17	46	17	+	63%
23 – A major strength of the Mini-CEX is its ability to allow direct, real-time observation of resident-patient interactions.	2	10	12	48	25	+	73%
24 – The main strength of the Mini-CEX is its evaluative capacity.	2	23	25	43	5	_	25%
25 – The main strength of the Mini-CEX is its ability to provide feedback.	2	2	15	43	35	+	78%
26 –The Mini-CEX results could be incorporated as part of the resident’s final annual assessment.	12	7	4	48	4	+	52%
27 – The results of the Mini-CEX could be analyzed to provide additional overall feedback at the end of residency training.	7	7	17	38	28	+	66%
Total “correct” responses (questions 11–27)							58%
Total “correct” responses							68%

SD, Strongly Disagree (%); D, Disagree (%); NaNd, Neither agree nor disagree (%); A, Agree (%); SA, Strongly Agree (%); CR, Responses Considered “Correct”; CRR, Correct Response Rate (%); (+) Agree or strongly agree; (-) Disagree or strongly disagree.

**Table 5:** Classification of Google Forms responses to Mini-CEX questions according to years of preceptorship and residency year.

	Mean score Q1‒Q10	SD	p-value	Mean score Q11‒Q27	SD	p-value	Overall mean	SD	p-value
**Preceptors**	86	3.5	p = 0.0545^a^	77.3	4.8	**p = 0.006**^a^	80.5	3.9	**p = 0.0006**^a^
**Residents**	76.8	3.5		52.2	4.6		61.3	3.8	

**Preceptors with < 10-years of preceptorship**	85	5	p = 0.35^a^	76.5	4.2	p = 0.62^a^	79.7	2.4	p = 0.35^a^
**Preceptors with ≥ 10-years of preceptorship**	86.4	4.5		77.5	6.4		80.8	5.3	

**Faculty members**	97.5	2.5	**p = 0.0104**^a^	88.3	3.4	p = 0.12^a^	91.7	1.8	**p = 0.0177**^a^
**Preceptors**	81.8	4		73.3	6		76.5	4.8	
									
**First- and second-year residents**	81.1	5.1	p = 0.7^a^	59.5	5.7	p = 0.65^a^	67.5	4.8	p = 0.8^b^
**Third-year residents**	70	11.5		49	10.1		56.8	10.6	
**Fellows**	77	3.7		47.7	8.6		58.5	6.1	

Mean score Q1‒Q10: Mean score for the first 10 questions.

SD, Standard deviation.

Mean score Q11‒Q27: Mean score for the last 17 questions.

a Kruskal-Wallis test with Dunn’s post hoc test.

b Mann-Whitney test.

For the 10 knowledge items (institutional procedures/constructs), the mean correct-response rate was 87%, with all groups exceeding 70%. The lowest correct-response rate was observed for the statement that the preceptor should choose the timing, competency, and patient for the Mini-CEX (45% correct), which conflicts with institutional guidance that these selections should be made by the resident. The highest correct-response rate was observed for the statement describing feedback as specific, balanced, and free of personal judgment (91%).

For the 17 perception items, the mean agreement with expected educational impact was 58%. Neutral responses were most frequently expressed for the statement that Mini-CEX improves the teaching-learning process (28% neutral). The highest agreement concerned feedback as the main strength of the Mini-CEX (78%). In analyses restricted to perception items, preceptors reported higher perceived impact than residents (p = 0.006) (Table 5).

In open-ended responses, prior exposure to the Mini-CEX was reported by 24% of residents and 33% of preceptors. Participants identified feedback as the most frequently cited advantage (40%), followed by the perception that the tool is practical and educational (37%). The most common disadvantages were perceived interference with outpatient workflow (36%) and concerns about assessment adequacy (36%), including selective sampling of subspecialties or preferred preceptors and the punctual nature of observations. The most frequent suggestions were improving feedback quality, allocating dedicated time for Mini-CEX completion, and refining implementation rules (e.g., clearer expectations regarding frequency, timing, and sampling).

## Discussion

The Mini-CEX is one of the most widely used WBA tools, combining direct observation with formative feedback.^1^^,^^2^ However, evidence in Otorhinolaryngology remains limited, restricting specialty-specific benchmarking and implementation guidance.^10^, ^11^, ^12^, ^13^ Our study expands this evidence by describing the feasibility of Mini-CEX implementation, assessment patterns, score distribution, and feedback documentation in an outpatient Otorhinolaryngology residency program.

According to Norcini and Burch,^7^ the educational value of the Mini-CEX depends on repeated observations across diverse clinical contexts, multiple assessors, and specific, actionable feedback. These principles help explain our main findings: the lack of complete feedback, the need for rater training and protected assessment time, and the difficulty in achieving the target of four encounters per resident during the early implementation phase.

Although feedback was documented in 90% of encounters, only one assessment (2.5%) met the predefined criteria for complete feedback, highlighting a major implementation gap. This finding is consistent with the implementation challenges reported in recent Mini-CEX studies.^25^, ^26^, ^27^ In a busy emergency department, Chang et al. found complete feedback in only 22.9% of 1,101 assessments, which the authors also considered suboptimal.^27^ Similarly, a recent Otorhinolaryngology study reported limited rates of complete feedback (12%), although it was conducted in an undergraduate setting.^11^ Likewise, Moe et al.^17^ and Martinsen et al.^28^ showed that the educational value of the Mini-CEX depends primarily on feedback quality rather than numerical ratings or the number of encounters. The finding that 68% of the feedback provided by residents was also non-specific further suggests an institutional deficit in feedback culture and feedback literacy. Consistently, feedback adequacy was not associated with preceptor experience (p = 0.72), indicating that this challenge reflects program design rather than individual assessor performance. Taken together, the available evidence suggests that low feedback adequacy reflects a recognized challenge in Mini-CEX implementation, which may be exacerbated by an unstructured feedback template, limited rater training focused on feedback delivery, and high patient volume.^25^, ^26^, ^27^^,^^29^

Although most encounters involved cases of moderate or high complexity, feedback documentation remained poor. While greater case complexity has been associated with richer feedback,^25^, ^26^, ^27^ our findings indicate that simply providing dedicated feedback fields does not ensure specific or actionable documentation. In busy outpatient clinics, verbal feedback may occur without being documented; nevertheless, written documentation remains essential for longitudinal CBME.

Subspecialty sampling was heavily skewed toward otology, likely because otologic examinations are perceived as more straightforward and time efficient. In a high-volume outpatient setting, this may also explain why multiple competencies were assessed in a single encounter and why 22% of forms contained incomplete case documentation. Together, these observations suggest that workflow constraints also played a substantial role in Mini-CEX implementation.

Nearly half of all ratings (46%) were classified as “above expectations”, suggesting possible rater leniency, ceiling effects, and/or selective case sampling. Previous studies indicate that broader sampling and improved rater calibration enhance assessment reliability and reduce score inflation.^4^^,^^17^ Residents more frequently selected less experienced preceptors, who assigned lower ratings.^27^ Although causality cannot be established, this finding underscores the importance of diversifying assessors and providing shared calibration and feedback training to promote consistent performance standards.^30^

The Mini-CEX captured the expected progression from first- to later-year residents, although no differences were observed between second- and third-year trainees.^1^^,^^10^^,^^12^ This may reflect both performance convergence and score inflation. For senior residents, complementary assessment methods such as Direct Observation of Procedural Skills (DOPS) may better capture higher-level competencies and broaden competency evaluation and specifically assess practical and surgical skills.^5^^,^^15^ Given the procedural and surgical nature of Otorhinolaryngology, the DOPS can complement the Mini-CEX by providing direct assessment of technical performance during surgical and other procedural activities.^31^

Survey responses demonstrated good understanding of institutional Mini-CEX procedures but only modest perceptions of its educational impact, particularly among residents. This perception may reflect the limited educational benefit derived from the current implementation, likely related to incomplete feedback provided by preceptors and the lack of protected time to conduct Mini-CEX assessments. These same factors may also have contributed to the difficulty in achieving the institutional target of four Mini-CEX encounters per resident. Together, these findings reinforce that the educational value of the Mini-CEX depends on repeated observations, meaningful and actionable feedback, and integration within a programmatic assessment framework rather than on isolated assessment encounters.

### Limitations

This single-center study was conducted in a tertiary Otorhinolaryngology outpatient service, limiting generalizability to other residency programs. The small and uneven sample (40 encounters involving 22 residents and 17 preceptors) reduced statistical power and subgroup precision, while the target of four encounters per resident per year was not achieved. Subspecialty sampling was skewed toward otology, limiting extrapolation to less frequently represented areas. The study questionnaire was developed specifically for this project and lacked prior validation, although it demonstrated excellent internal consistency (Cronbach’s α = 0.96). Feedback quality was assessed only from written documentation, so verbal feedback may have been underestimated. Finally, the retrospective design precludes exclusion of residual confounding. Despite these limitations, the consistency of our findings with previous Mini-CEX studies suggests that the observed feedback inadequacy, rating leniency, and uneven sampling reflect implementation challenges rather than isolated findings.

### Operationalized recommendations for implementation

Rather than general recommendations, we propose the following implementation strategies, several of which have already been adopted in our program.

1) Protected assessment time: allocate a dedicated 20-minute slot (10-minutes for observation and 10-minutes for feedback and documentation) within the outpatient schedule.
2) Structured feedback template: replace the single free-text field with three mandatory sections (“Strengths”, “Areas for improvement”, and “Action plan”) to promote complete, actionable feedback.
3) Rater development: provide annual calibration workshops, including onboarding for new preceptors, focused on delivering actionable feedback rather than scoring alone.
4) Deliberate sampling: ensure balanced sampling across subspecialties and assessors through predefined monitoring targets.
5) Completeness monitoring: perform routine administrative checks and issue automated reminders for incomplete forms.
6) Complementary assessment: incorporate the DOPS and entrustment-based assessments, particularly for senior residents, to broaden competency evaluation and specifically assess practical and surgical skills.^5^
7) Rating scale revision: replace the current three-point scale with the original nine-point Mini-CEX scale to improve score discrimination and comparability with previous studies.^1^

Items (1–3) should be prioritized, as they directly address the main barriers identified in our study: time constraints, unstructured feedback, and limited rater training.

## Conclusions

This early implementation evaluation suggests that Mini-CEX use in an Otorhinolaryngology outpatient residency program is feasible and can detect expected progression in performance across training years. However, the process also revealed substantial opportunities for improvement, particularly regarding subspecialty sampling balance, potential score inflation, and ‒ most importantly ‒ the documentation of specific, actionable feedback. Strengthening rater development, protecting time for observation and feedback, instituting deliberate sampling across subspecialties and assessors, and progressively increasing the number of observed encounters is recommended to maximize the educational impact of this competency-based assessment strategy.

## ORCID ID

Carolina Sponchiado Miura: 0000-0002-2874-7689

Valdes Roberto Bollela: 0000-0002-8221-4701

## Declaration of Generative AI and AI-assisted technologies in the writing process

During the preparation of this work the author(s) used ChatGPT in order to translate. After using this tool/service, the author(s) reviewed and edited the content as needed and take(s) full responsibility for the content of the published article.

## Funding

Financial support from CNPq Grant (PQ-1D) under grant number 302384/2022-7 to FCPV.

## Data availability

The authors declare that all data are available in repository.

## Declaration of competing interest

The author(s) declared no potential conflicts of interest with respect to the research, authorship, and/or publication of this article.
