# Supplementary material for: Early experience with Mini-CEX in otorhinolaryngology residency: feasibility, feedback quality, and perceived educational impact
Source: Braz J Otorhinolaryngol. 2026 Oct 31;93(1):101944. doi: 10.1016/j.bjorl.2026.101944 (PMC13743947; doi:10.1016/j.bjorl.2026.101944)
Supplement: Supplementary file 1 [file mmc1.docx]

BJORL-D-26-00341_Supplementary Material

**Appendix 1** Feedback adequacy rubric.

| **Effective Feedback** | **Direct** | **With positive points** | **With negative points** | **with an improvement plan** |
| --- | --- | --- | --- | --- |

Effective Feedback: Direct feedback with positive and negative points and an improvement plan.

**Appendix 2**The 27-item online questionnaire (Google Forms) and its corresponding answer key.

| **Questions** | **Responses considered “correct”** |
| --- | --- |
| 1 – Each mini-CEX assesses the resident’s ability to perform interviewing, physical examination, or patient counseling with a real patient. | + |
| 2 – The mini-CEX can assess professionalism, clinical reasoning, organization/efficiency, and overall clinical competence. | + |
| 3 – The resident is expected to request a preceptor to conduct the mini-CEX assessment. | + |
| 4 – The resident is expected to organize their schedule to maintain the mini-CEX assessment frequency as stipulated in the resident handbook. | + |
| 5 – The preceptor is expected to choose the timing, the competency, and the patient for assessment using the mini-CEX. | _ |
| 6 – The resident is expected to organize their assessments to complete mini-CEX evaluations covering the three competencies (interviewing physical examination, and patient counseling), with different evaluators and across different subspecialties. | + |
| 7 – The resident is expected to exercise judgment in selecting the most appropriate time to request an assessment so as not to disrupt service workflow. | + |
| 8 – The preceptor is expected to decide whether or not to provide feedback for each mini-CEX assessment. | _ |
| 9 – Feedback is expected to address both positive and negative aspects, be specific, direct, and assertive, avoid personal judgments, and remain independent of case severity or complexity. | + |
| 10 – During feedback, the preceptor is expected to encourage initial resident self-assessment, provide guidance for improvement, and solicit feedback on their own evaluation. | + |
| 11 – The feedback provided by the mini-CEX helps residents improve their knowledge, skills, and attitudes. | + |
| 12 – Mini-CEX assessments can identify gaps in the teaching–learning process and guide strategies to address these challenges. | + |
| 13 – Mini-CEX assessments can help residents plan their continuous knowledge acquisition, thereby supporting the development of qualified professionals. | + |
| 14 – The mini-CEX improves the teaching–learning process. | + |
| 15 – The use of the mini-CEX should be encouraged. | + |
| 16 – The mini-CEX should be replaced by another assessment method. | _ |
| 17 – The mini-CEX is a practical assessment tool. | + |
| 18 – The mini-CEX is an easy-to-use assessment tool. | + |
| 19 – The mini-CEX is an efficient assessment tool. | + |
| 20 – The use of the mini-CEX contributes to improving the resident–preceptor relationship. | + |
| 21 – The use of the mini-CEX helps residents prepare for theoretical examinations. | _ |
| 22 – The use of the mini-CEX influences the development of professionalism among residents. | + |
| 23 – A major strength of the mini-CEX is its ability to allow direct, real-time observation of resident–patient interactions. | + |
| 24 – The main strength of the mini-CEX is its evaluative capacity. | _ |
| 25 – The main strength of the mini-CEX is its ability to provide feedback. | + |
| 26 –The mini-CEX results could be incorporated as part of the resident’s final annual assessment. | + |
| 27 – The results of the mini-CEX could be analyzed to provide additional overall feedback at the end of residency training. | + |
| Total “correct” responses (questions 11‒27) | |
| Total “correct” responses | |

CR, Responses considered “correct”; (+) Agree or strongly agree; (-) Disagree or strongly disagree.
